# Implementation of a passive bi-articular ankle–knee exoskeleton during maximal squat jumping

**DOI:** 10.1098/rsos.240390

**Published:** 2024-07-31

**Authors:** Logan Wade, Glen Lichtwark, Dominic Farris

**Affiliations:** ^1^ Department for Health, University of Bath, Bath, UK; ^2^ School of Human Movement and Nutrition Sciences, The University of Queensland, Brisbane, Queensland, Australia; ^3^ School of Exercise and Nutrition Sciences, Queensland University of Technology, Brisbane, Queensland, Australia; ^4^ Public Health & Sport Sciences, University of Exeter, Exeter, UK

**Keywords:** assistive device, soft exoskeleton, elastic, gastrocnemius

## Abstract

Owing to the unexplored potential to harness knee extension power during jumping, the current study aimed to examine how joint mechanics were altered with a biologically inspired, passive bi-articular ankle–knee exoskeleton, which could potentially facilitate greater jump height by increasing work production about the knee and ankle. Twenty-five participants (16 males and 9 females, 175.2 ± 8.2 cm, 72.9 ± 10.3 kg, 24.0 ± 3.4 years) performed maximal squat jumping with and without the exoskeletal device and we compared jump height, joint moment and joint work of the lower limbs. Despite a low exoskeleton stiffness and therefore a limited capacity to store energy, the bi-articular device resulted in decreased jump height (1.9 ± 3.1 cm, *p* = 0.006), decreased net work about the knee (0.23 J/kg, *p* < 0.001) and no increase in ankle joint work (*p* = 0.207), compared with jumping with no exoskeleton. Based on our findings, to mimic unassisted ankle joint moment profiles, a future bi-articular device would need increased elastic element slack length, greater stiffness and a larger moment arm about the ankle. Future designs could also employ attachment sites that have minimal overlying soft tissue, such as the pelvis, to improve comfort of the device.

## 1. Introduction

Integration of exoskeletal devices, which attach to the human body to alter or enhance movement, has the potential to improve human performance in leg extension tasks such as jumping [1,2] or heavy lifting [3]. Functional human movement requires exoskeletal force to be applied at the correct instant and over the optimal period of time [4–6], so as not to disrupt joint mechanics such as a proximal-to-distal sequence of leg extension [7]. Much of the research into exoskeletal design has surrounded mono-articular devices, where only a single joint is directly affected by the device [1,8,9]. However, bi-articular exoskeletons, which act over two or more joints, could play an important role in transferring power from proximal joints to distal joints and fine-tuning torque generation, similar to the roles of bi-articular muscles [10–14]. Biologically inspired, bi-articular exoskeletal devices could have a substantial impact on force production, enabling heavy actuators to be located more proximally on limbs. An ankle–knee bi-articular device is of particular interest owing to the important role bi-articular plantar flexors play in take-off during jumping, and the potential for power transfer from the knee to the ankle [11,12,15].

Researchers have explored applications of bi-articular actuators to increase jump height in robots [10], demonstrating improved results with bi-articular actuators attached to both thigh and shank segments [16]. Another group used a compliant robotic leg to demonstrate improved jumping efficiency with bi-articular actuators [17]. Unfortunately, we are currently unaware of any human exoskeletons that have mimicked the bi-articular gastrocnemius muscle for use with jumping. Two studies have used powered mono-articular ankle exoskeletons during jumping to increase ankle torque [18,19], with one study reporting an increase in jump height of 3.1 cm [18]. Increasing jump height using an exoskeletal device is further complicated when devices add mass to the body, which may counteract the benefit derived from the assistance of the exoskeleton [20–24]. To reduce added mass on distal limbs, devices might be constructed with elastic structures to store and return energy [9,25–28], which provides a simple but effective method for applying an external force to the wearer with minimal added mass [9,28,29]. Only one study has examined jumping with a passive device [1]. Using a mono-articular knee exoskeleton they initially observed a decrease in jump height (2.2 cm lower), however after running optimization simulations they then trained their users to jump with a new technique [30], observing an increase in jump height (2.7 cm higher) with a much deeper countermovement depth [1]. However, we believe a bi-articular device could have substantial potential to increase jump height given it would directly impact two joints, without requiring such high forces as Ben-David *et al*. [1]. On the other hand, by impacting two joints there is also greater potential to negatively impact joint mechanics, thus, research is needed to understand how incorporating a bi-articular structure within exoskeletal designs will impact torque output about the lower limbs.

To increase jump height, a passive bi-articular device that mimics that gastrocnemius would resist knee extension to store elastic energy, which could then be used to assist with ankle plantar flexion. During maximal effort movements, it is generally assumed that muscles are contracting maximally. Thus, there would be limited capacity to perform additional work by storing energy in an elastic tendon during maximal jumping. However, a previous study examined knee extension moment during the upward phase of explosive squat weight-lifting, identifying that the knee extension moment did not increase with added weight as was done in the hip and ankle [31]. It was suggested that the knee extensors were limited by constraints requiring them to maintain control of the ground reaction force (GRF) vector orientation, which is determined by balancing knee extension moments against hip extension and ankle plantarflexion moments. The result was a sub-maximal effort about the knee joint despite performing a maximal effort movement, indicating the potential to draw on a reserve of knee extensor force during maximal leg extension tasks [31]. The bi-articular gastrocnemius facilitates power transfer from knee extensor muscles by simultaneously resisting knee extension and generating ankle plantarflexion moments [32]. Therefore, an exoskeleton that mimics gastrocnemius function might be able to harness reserves of knee extensor moment, without disrupting joint mechanics during explosive leg extension, transferring this energy down to assist with ankle plantar flexion through implementation of a bi-articular exoskeleton. Ultimately this exoskeletal design might increase maximal jump height by tapping into a reserve of muscle capacity without requiring any external power input.

**Figure 1.**
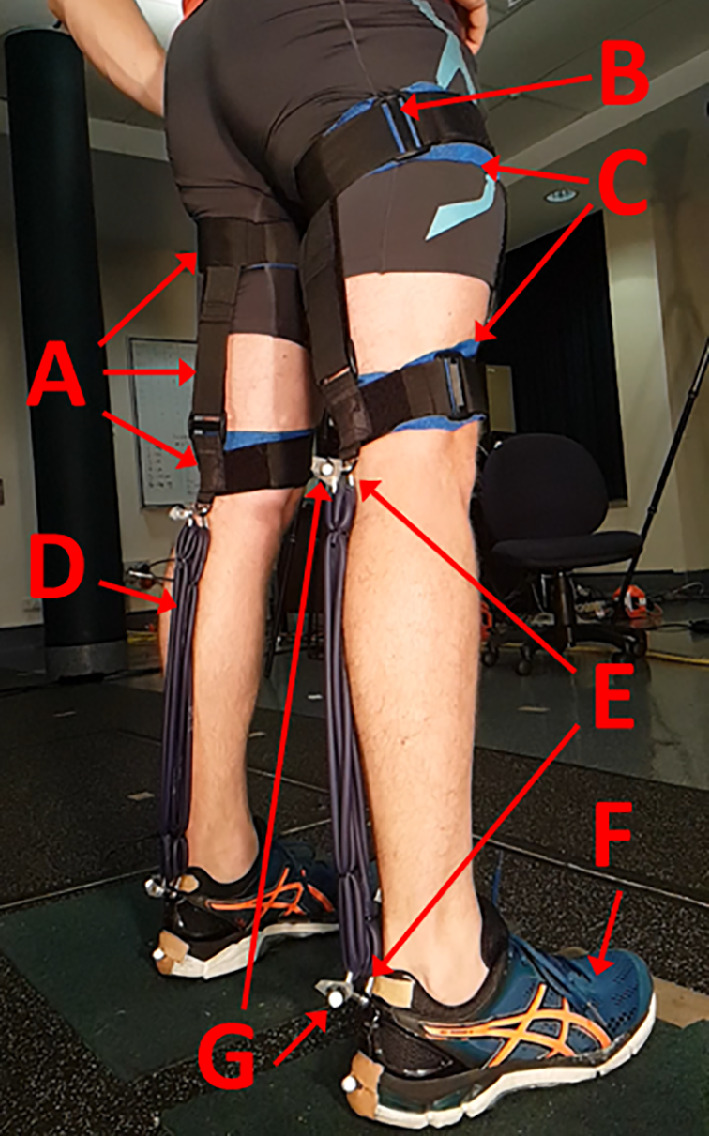
The exoskeleton consisted of velcro straps (A), O-rings (B), self-adhesive bandages to improve comfort (C), elastic tubing (D), D-shackles and D-rings (E), shoes (F) and markers placed on either end of the elastic tubing to measure exoskeleton length during jumping (G).

We designed a passive bi-articular, elastic exoskeleton using lightweight textile materials [24,33] and latex elastic tubing to store and return elastic energy (figure 1). The intention of this device was to harness the untapped force capacity of the knee extensors without altering net knee moments, resisting knee extension to store elastic energy and then transferring this additional energy distally to assist with ankle plantar flexion and increase jump height. Therefore, this study aimed to examine how kinematics and kinetics of the hip, knee and ankle joints were altered when maximally jumping with an elastic bi-articular exoskeleton compared with normal jumping (no exoskeleton). We hypothesized that when using the bi-articular device, maximal jump height would be increased, owing to an increased biological knee moment storing energy in the elastic exoskeleton, which would be transferred down to the ankle to increase net work about the ankle late in push-off.

## 2. Methods

### 2.1. Participant recruitment

Twenty-seven participants (18 males and 9 females, 175.6 ± 8.3 cm, 73.0 ± 10.1 kg, age = 24.0 ± 4.0 years) gave written informed consent to participate in this study. Ethical approval was granted by the institutional ethics review committee at The University of Queensland (HMS15/1106) and participants were recruited between April 2017 and October 2017. Owing to missing force plate data from two participants, data from 25 participants were included in the final analysis (16 males and 9 females, 175.2 ± 8.2 cm, 72.9 ± 10.3 kg, 24.0 ± 3.4 years). Participants initially attended the lab to be measured for their personalized exoskeleton slack lengths (measurement session). Participants returned to the lab for a familiarization session, after which participants again returned on a separate day to perform data collection with the bi-articular exoskeleton. To be included in this study, participants had to be aged between 18 and 35 years old, did not need to be trained jumpers, and had to be free from any injuries or clinical disorders that would impact their maximal jumping ability, to ensure they could perform maximal jumping with minimal risk of injury. Participants were required to refrain from eating within 1 h prior to jumping sessions and were not allowed to have performed strenuous physical activity the day before jumping sessions.

### 2.2. Exoskeletal design

The primary goal in designing the bi-articular exoskeleton was to create a device that was lightweight and could be adjusted to fit all participants. Velcro straps were sewn together, consisting of two horizontal bands circling around the top and bottom of the thigh (figure 1), which were supported by two vertical straps (one anterior and one posterior) and made adjustable through the use of plastic O-rings (figure 1*b*). The rationale for having two horizontal bands connected by vertical bands is that at 90° knee angle, all the load will be transmitted to the body through the lower horizontal band. However, as the knee is extended and the loading becomes more parallel with the thigh, this will pull the lower horizontal velcro band down the leg, which would require this lower band to be incredibly tight to resist this loading direction. Therefore, the upper band is placed above the muscle mass of the thigh and connected to the lower band to stop the lower band from moving as the knee is extended. This enables both bands to be attached with enough pressure to resist slippage, but not so much that it overly constricts the thigh and hinders muscle contraction. Attachment points for the elastic tendon used metal D-rings, one attached to the velcro strapping and one attached to the heel of a shoe provided for the participant (figure 1). The bi-articular device implemented a high strap above the thigh muscle mass, which was tethered to support a low strap just above the knee, where the proximal D-ring was attached (figure 1*b*). Self-adhesive bandages (Medichill, WA, Australia) were placed underneath each horizontal velcro strap to improve comfort (figure 1*c*). TheraBand latex resistance tubing (TheraBand Blue, TheraBand, OH, USA) was measured and cut to subject-specific lengths, before being looped one or two times and secured using zip ties (figure 1*d*). Two sets of single loops and one set of double loops were worn at the same time, which increased the stiffness of the tubing by a factor of eight. The chosen stiffness was easily tolerable by the participants and was the highest stiffness that could be resisted by the velcro strapping without tearing free. The tubing was attached to the proximal and distal D-rings by the use of D-shackles (figure 1*e*). The distal D-ring was attached to the heel of the shoe worn in the study (Asics Gel Pulse—figure 1*d*). The exoskeleton can therefore be thought of consisting of three parts; shoes, TheraBand tubing and thigh straps. The shoes weighed 571–683 g depending on the participant’s shoe size. TheraBand tubing weighed 152–214 g depending on length required for each participant. Thigh straps weighed 163 g. Therefore, the total weight of the exoskeleton was 886–1060 g. Testing was performed with shoes and thigh strapping in place for both conditions. Therefore, the difference in weight between the exoskeleton and no exoskeleton condition was 152–214 g.

### 2.3. Measurement session

**Figure 2.**
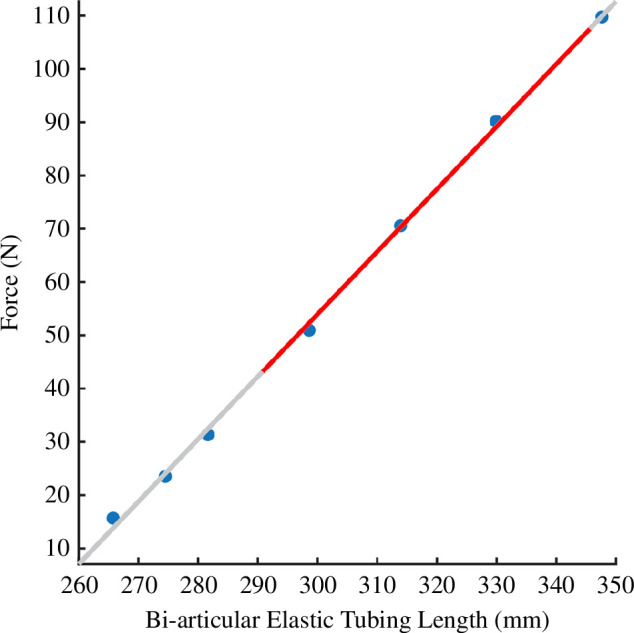
Example exoskeleton force–length relationship for a single participant. Blue dots indicate the length of the exoskeleton when the corresponding weight was hung from the device. Red overlay of the linear regression line indicates the portion over which the exoskeleton operated when worn by the participant.

Prior to testing, participants attended the lab for a measurement session. The exoskeleton was attached to the participant using velcro, which enabled personalized fitting of the horizontal and vertical velcro straps to account for different thigh thicknesses (figure 1*a*). The lower horizontal velcro straps were placed so that the posterior aspect of the velcro where the TheraBand attaches (figure 1*e*) was flush with the back of the calf when squatting with a 90° knee angle. The upper velcro strap was placed high on the thigh, above the muscle mass to take most of the load when the leg was fully extended. The upper and lower horizontal bands were connected using two vertical bands. Slack length of the TheraBand tubing was measured as the distance between the proximal and distal D-shackles when attached to the D-rings, while participants held the squatted position at a knee angle of 90°. This slack length was chosen to ensure that minimal energy was stored in the tendon when the person was squatting prior to push-off. Textile exoskeletons may suffer from displacement of the fabric during loading of the exoskeleton, which may alter the stiffness of the system as a whole and influence energy storage. To limit this effect, the velcro straps were constantly loaded by further shortening the measured slack length by 7.5%, which also ensured that the exoskeleton only operated on the linear portion of the elastic force–length relationship (figure 2). After each experimental session, tendon stiffness and slack length were calculated by applying a linear regression equation to the measured exoskeleton lengths when known weights were suspended from the elastic tubing. Calculation of exoskeleton force was performed using measured exoskeleton lengths during experimental data collection and the participant-specific linear regression equation (figure 2).

### 2.4. Familiarization session

**Figure 3.**
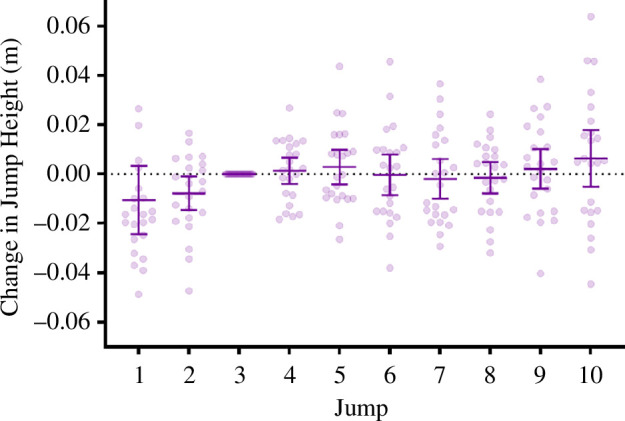
Familiarization jump height data for all participants using the bi-articular exoskeleton for their first 10 jumps. Mean ± s.d. are overlayed on top individual data. Jump height was normalized to the third jump for each participant.

Participants did not practice jumping with the exoskeleton prior to the familiarization data collection. An eight-camera, three-dimensional optoelectronic camera system (Oqus, Qualisys, AB, Sweden) was used to capture kinematic data (200 Hz) and was calibrated as per Qualisys’ standard protocol. Two force plates (OR6-7, AMTI, MA, USA) were used to collect kinetic data (1000 Hz), with one foot placed on each plate. To minimize setup time, only two markers were required to calculate jump height in combination with the force plates, with these markers placed on the left and right anterior superior iliac spine [34]. Upon arrival to the lab, the participants were attached with the velcro straps for the exoskeleton and optoelectronic markers, after which participants performed a self-selected warm-up to ready themselves for maximal jumping. Once warmed up, the personalized elastic TheraBand tubing was attached to the velcro strapping. Finally, participants performed 10 maximal squat jumps with the bi-articular exoskeleton, with at least 90 s between jumps. During jumping, participants first assumed a quiet stance position, after which they descended to a squat position with their knees at 90°. This was controlled by the application of a horizontal rope, installed posterior to the participant, which touched the back of their thighs at the correct depth. After holding this squatted position for 2 s, participants jumped maximally. Participants were instructed not to perform a countermovement (i.e. to only move in the upwards direction), and were given an unweighted vest with instructions to lock their hands onto the side of the vest to ensure that arms were not used during jumping. Statistical analysis of the familiarization session was conducted to determine if performing more than three jumps was needed to obtain a maximal jump height in the experimental session. Familiarization jump height data were analysed using a one-way repeat measures ANOVA to examine if there was a main effect of performing additional jumps with the exoskeleton on jump height. Post hoc analysis was performed comparing all jump heights relative with the third jump for each participant (figure 3). Jump heights collected during the familiarization session indicated that there was no significant main or interaction effect of performing more than three jumps (figure 3) and therefore, three jumps were sufficient to obtain a maximal jump height with the exoskeletal device.

### 2.5. Experimental session

Upon arrival at the lab for data collection, the exoskeleton velcro strapping (figure 1) and markers were affixed to the participant. Participants then performed the same self-selected warm-up as in the familiarization session to ready themselves for maximal jumping. For the experimental condition, the personalized elastic TheraBand tubing was attached to the velcro strapping. In the control condition, participants still wore the velcro straps and shoes, but no elastic tubing was attached. Once setup for each condition was complete, participants performed two sets of 10 preferred double-leg hops and two submaximal jumps to acclimate to the new condition. Participants then performed three maximal jumps with at least 90 s of rest in between jumps using the exoskeleton (exoskeleton condition) and without the exoskeleton (control condition), in a block randomized order. During the experimental session, participants perform the jump in the exact same method as the familiarization session, descending to a 90° knee angle and holding the squatted position for two sections before performing a maximal jump with hands locked onto the side of their vest.

### 2.6. Data collection and analysis

Using the same hardware setup as in the familiarization session, kinematic and kinetic data were collected. During experimental data collection, reflective markers (12 mm) were placed on anatomical landmarks for static and dynamic measures. We used a modified version of the marker set included with the Hamner *et al.* [35] and Rajagopal *et al*. [36] model, with markers placed on the acromion process of the left and right shoulders, suprasternal notch of the manubrium, C7 vertebrae, left and right posterior superior iliac spine, left and right anterior superior iliac spine and along the left and right iliac spine directly superior to the greater trochanter. Markers were placed on both legs at the medial and lateral joint centre of rotation of the knee, medial and lateral malleolus of the ankle with clusters of four markers on rigid plates placed on the lateral side of the shank and thigh, midway between joints using velcro straps. Markers for both feet were placed on the shoes over the posterior aspect of the calcaneus, metatarsal phalangeal joints 1 and 5, and the distal phalanx of the first toe. Custom shoulder (acromion process) markers were built to track shoulder movement while wearing a weight vest. Additional markers were placed on the superior and inferior ends of the exoskeleton tubing of each leg to measure exoskeleton length change. Jump heights in the familiarization and experimental sessions were calculated using the hybrid method previously described by Wade *et al*. [34], which combines flight distance calculated using a force plate, with heel-raise distance calculated using three-dimensional motion capture from markers placed on the pelvis. Only the maximal jump height trial for each condition was used for data analysis as this study’s aim was to determine how the exoskeleton altered the kinematics and kinetics of maximal jumping and all other jumps by each participant lower than the maximal are by definition, sub-maximal jump heights. Some participants subconsciously performed a slight countermovement during push-off to jump maximally, however, jumps were excluded if subjects performed a countermovement that resulted in their GRF reducing by more than 20% bodyweight. Post data collection, markers were labelled in Qualisys Track Manager (Qualisys, Gothenburg, Sweden) and exported to OpenSim [37].

A generic OpenSim model, previously described by Hamner *et al.* [35], was modified to remove the upper arms, forearms and hands, with their masses added to the head and trunk segment, and the cervical joint locked. Segment lengths, inertial parameters and masses were scaled according to their respective ratio within the generic model, keeping distribution of masses the same as in the generic model. Once scaling of the model was performed, inverse kinematic analyses were completed using a weighted least-squares fit of the generic model markers to the experimental markers during jumping trials [37]. Inverse kinematic results for joint angles were then combined with GRF data in an inverse dynamics analysis to calculate joint moments of the ankle, knee and hip. Filtering was performed on raw marker positions in OpenSim prior to inverse kinematics and ground reaction forces prior to inverse dynamics calculations, with filtering in both instances completed using a 25 Hz second-order two-way Butterworth filter. Inverse kinematics and dynamics results for each trial were imported into MATLAB (MathWorks, Natick, MA, USA), where a custom script used the central difference technique to differentiate joint angles. Resulting joint velocities were multiplied by joint moments to calculate joint powers.

Using a custom MATLAB script, joint power time-series data were trimmed from the start of push-off through to take-off. Event timings were calculated using GRF data when determining jump height [34]. Left and right joint powers were summed at the hip, knee and ankle, and integrated to calculate hip, knee and ankle joint work. Net work was the time integral of all power values, positive work was the time integral of all positive power values and negative work was the time integral of all the negative power values, for each joint from the start of push-off until take-off. Ankle and knee exoskeleton moment arms (distance between the muscle line of force and the joint centre of rotation) for each leg were calculated at each instant throughout the jumping movement. Exoskeleton moment arms were defined as the shortest distance between the ankle joint axis (median point between malleoli markers) or knee joint axis (median point between knee joint line markers), and the line of action of the exoskeleton calculated using the inferior and superior exoskeleton markers. Two centimetres were then subtracted from the knee and ankle exoskeleton moment arm to account for exoskeleton markers being placed 2 cm posteriorly from the midline of exoskeleton line of action. Exoskeleton elastic element force was multiplied by the exoskeleton moment arm to calculate exoskeleton moment about the ankle and the knee. Owing to the exoskeleton assisting plantar flexion and resisting knee extension, calculation of the moment produced by the muscles alone is required to determine changes in biological force production about the ankle and knee. Flexion–extension moments about the hip, knee and ankle were calculated through inverse dynamics analysis using the OpenSim inverse dynamics tool. As the exoskeleton was aligned to contribute to (plantar)flexion of the knee and ankle, only flexion–extension joint moments were examined. Ankle and knee biological moments were then calculated by subtracting the exoskeletal moment from the net joint moment at the knee and ankle at every sampling point. Net moment, biological moment and exoskeleton moment were then averaged between the left and right legs. Angular impulse was calculated for each joint (ankle, knee and hip) as the time integral of joint moments. Net angular impulse was the time integral of net moment values across the entire movement for each joint, positive angular impulse was the time integral of all positive moment values, and negative angular impulse was the time integral of all negative values.

### 2.7. Statistical analysis

Statistical analysis was performed in GraphPad Prism 9 (GraphPad Software Inc, California, USA). Normality testing was performed in Prism (*t*-tests and ANOVA) or in the SPM1D toolbox. For *t*-tests, normality was assessed through agreement between D’Agostino–Pearson, Shapiro–Wilk and Kolmogorov–Smirnov normality tests, which all passed. For the ANOVAs, normality was assessed through visual inspection of QQ normality plots, which did not identify any violations of normality. As per GraphPad prism guidelines, sphericity was not assumed as these results included repeated measures, and therefore the Geiser–Greenhouse correction was used. Normality for SPM1D analysis used D’agnostino–Pearson normality test which all passed. A paired *t*‐test was used to compare jump height and summed joint work between the exoskeleton and control condition. A post hoc power analysis was performed using G*Power [38], based on the change in jump height between the control and exoskeleton conditions observed in this study (effect size = 0.606). Using an alpha of 0.05 and the sample size of *n* = 25, the power of this study was identified as 0.828, thus this study was sufficiently powered to examine differences between these two conditions. Because the bi-articular exoskeleton will directly affect both the knee and the ankle, and may indirectly influence the hip, two-way repeat measures ANOVAs were used to examine main (exoskeleton versus control) and interaction (two exoskeleton conditions × three joints) effect on individual joint-level outcome variables for the hip, knee and ankle. If a main effect of exoskeleton or interaction effect was found, then post hoc multiple comparisons were performed to examine the difference between each joint in the exoskeleton and control condition. One-dimensional statistical non-parametric mapping (SnPM1D) analysis [39] was performed in MATLAB (Mathworks, MA, USA) and employed on net and biological joint moments, relative to net moments in the control condition. To account for multiple comparisons across all statistical testing, a Benjamini and Hochberg false discovery rate (5%) approach was applied to all *p* values generated in this study [40]. This correction resulted in the alpha threshold decreasing from 0.050 to 0.033. Original *p* values are reported but only *p* values below the adjusted alpha threshold were considered significant.

## 3. Results

### 3.1. Jump height and joint work

**Figure 4.**
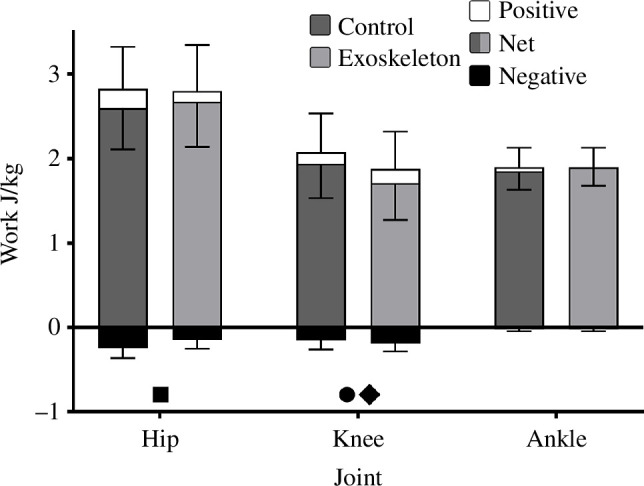
Mean net, positive and negative joint work (± s.d.) about the hip, knee and ankle in the exoskeleton and control conditions. Dark grey net work indicates the control condition while the light grey net work indicates the exoskeleton condition. ● Indicates a significant difference in net work. ♦ Indicates a significant difference in positive work. ■ Indicates a significant difference in negative work.

The bi-articular exoskeletal device resulted in a significant decrease in jump height by 1.9 ± 3.1 cm (*p* = 0.006). Summed joint work of the hip, knee and ankle was significantly lower (*p* < 0.001) with the exoskeleton (6.28 ± 0.77 J/kg) compared with the control condition (6.37 ± 0.66 J/kg). Across all joint level measures, which were examined using two-way repeat measures ANOVA (net, positive and negative work, and net, positive and negative impulse), there was a significant main effect of joint, as expected owing to the different roles of each joint (*p* < 0.001), as well as a significant interaction effect of joint and exoskeleton condition (*p* ≤ 0.015). There was only a significant main effect of exoskeleton condition in positive and negative work (*p* < 0.001). Post hoc analyses identify that when jumping with the exoskeleton, negative work about the hip was reduced by 0. 1 J/kg (*p* < 0.001), net and positive work about the knee was reduced by 0.23 and 0.20 J/kg, respectively (*p *< 0.001) with no change at the ankle (*p* > 0.068) compared with the control condition (figure 4).

### 3.2. Exoskeletal and biological moments

**Figure 5.**
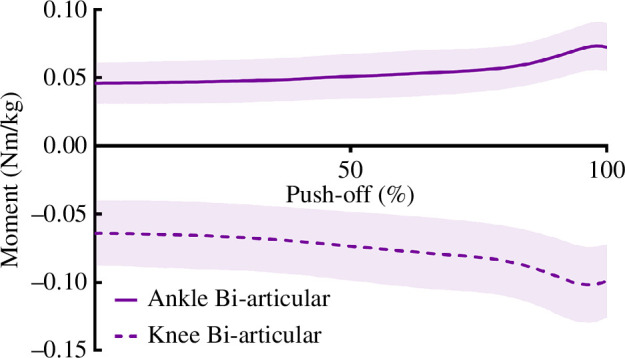
Mean bi-articular exoskeleton moment (± s.d.) about the knee and ankle. Time zero occurs at the point the toes leave the ground. Time-series data begins at the start of push-off and ends at toe-off.

**Figure 6.**
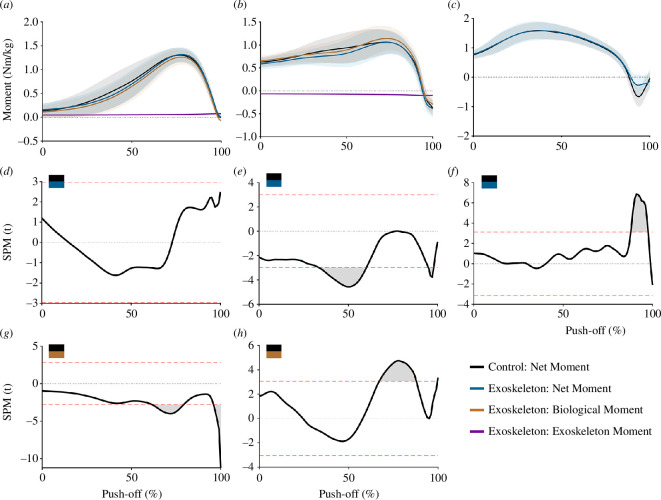
Mean time-series data of joint moment (± s.d.) about the ankle (*a, d, g*), knee (*b, e, h*) and hip (*c, f*). Graphs (*a*), (*b*) and (*c*) demonstrate the net joint moments, along with the biological and exoskeleton moments (ankle and knee only). Graphs (*d*), (*e*) and (*f*) present the 1D-SPM differences between the exoskeleton net joint moment and the control net joint moment for the ankle, knee and hip. Graphs (*g*) and (*h*) present the 1D-SPM differences between the exoskeleton biological moment and the control net joint moment for the ankle and knee. Time-series data begins at the start of push-off and ends at toe-off.

Mean exoskeleton stiffness was 1.07 ± 0.12 N/mm for the bi-articular device. The bi-articular exoskeleton moment about the ankle increased over the entire push-off phase owing to knee extension, before plateauing owing to ankle plantar flexion (figure 5). Moments generated by the exoskeletal device were substantially smaller than the net joint moment about the ankle and knee, opposing knee extension with only 10.5% of the mean net joint moment and assisting ankle plantar flexion with 8.3% of mean net joint moment (figure 6). Net and biological joint moments are described in figure 6, along with 1D-SPM results. Net knee moment was reduced midway through push-off (*p* < 0.001) and very shortly at the end of push-off (*p* = 0.011). Net hip moment was significantly increased immediately prior to take-off (*p* = 0.005) and there was no change in ankle net moment. Examination of biological knee moment demonstrated an increase during the peak period of the biological moment (approx. 65–85% of push-off, *p* < 0.001). There was a slight decrease in ankle biological moment during (approx. 60–80% of push-off, *p* = 0.006) and at the end of push-off (*p* = 0.002).

### 3.3. Joint angular impulse

**Figure 7.**
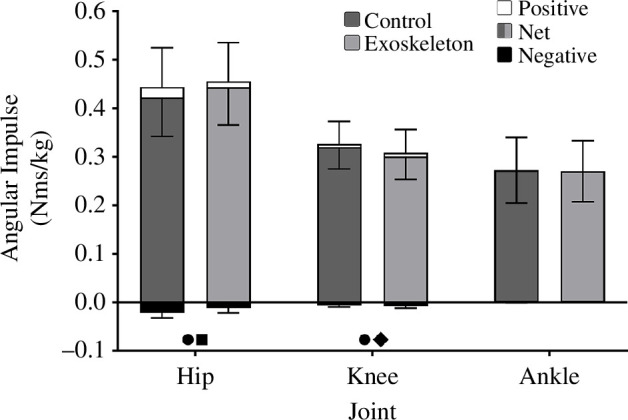
Mean net, positive and negative joint angular impulse of the bi-articular device (± s.d.). Dark grey net angular impulse indicates the control condition while light grey net angular impulse indicates the exoskeleton condition. ● Indicates a significant difference in net angular impulse. ♦ Indicates a significant difference in positive angular impulse. ■ Indicates a significant difference in negative angular impulse.

As mentioned previously, all joint level measures analysed using two-way repeated measures ANOVA (including joint angular impulse) had a significant main effect (*p *≤ 0.001) and interaction effect (*p* ≤ 0.015). Post hoc analysis identified that compared with the no exoskeleton condition, net angular impulse with the bi-articular exoskeleton (figure 7) increased about the hip (net = 0.021 Nms/kg, *p* = 0.001), as a result of a significant decrease in negative angular impulse (negative = 0.010 Nms/kg, *p* < 0.001). Net and positive angular impulse about the knee were significantly reduced (net = −0.019 Nms/kg, *p* = 0.006 and positive = −0.018 Nms/kg, *p* = 0.008) and there was no change in any angular impulse about the ankle (*p* > 0.240).

## 4. Discussion

The overall effect of the current exoskeletal design was a decrease in the overall summed net hip, knee and ankle joint work in the exoskeleton condition. This was mirrored by a decrease in maximal jump height relative to jumping with no exoskeleton (elastic bands removed only). The following paragraphs break down the work and moments produced about each joint to explain this decrease in jump height.

The exoskeleton, which resisted knee extension during push-off, did cause an increased biological knee extension moment relative to the control condition, albeit only for a brief period during push-off (65–85% of push-off, figure 6*b*). Ultimately, this increase was not sufficient to offset the lower net knee extension moment (biological + exoskeleton) during the middle of push-off (figure 6*b*), resulting in significantly reduced knee extension positive work, net work and angular impulse over the entire push-off, compared to the control condition (figures 4 and 7). Thus, increasing the biological knee extension moment may only be possible during mid-late push-off. The bi-articular exoskeletal device simultaneously assisted ankle plantar flexion but unfortunately did not result in any significant change in net moment, impulse or net work about the ankle compared with the control condition. Furthermore, during the period when the knee biological extension moment was increased (65–85% of push-off, figure 6*h*), the biological ankle plantar flexor moment was significantly reduced (60–80% of push-off, figure 6*g*). Thus, any increased energy transfer during this period appears to have been negated by a reduction in biological plantar flexor moment about the ankle, potentially to maintain a net ankle moment similar to the control condition (figure 6*d*). This may suggest a reluctance to increase net ankle moments by the plantar flexor muscles with an exoskeleton even in maximal effort movements, preferring instead to maintain net joint moment patterns produced in the control condition. Similar findings have been observed by previous studies using mono-articular ankle exoskeletons, which identified a reduced biological plantar flexor moment about the ankle during hopping and walking [5,9,26]. The overall decreased net extension moment about the knee, combined with the inability to increase the net moment about the ankle was likely the primary factors contributing to the decrease in jump height.

Despite no exoskeleton directly acting about the hip, the bi-articular exoskeleton resulted in a significant decrease in negative work and angular impulse about this joint (figures 6 and 7). Previous research has demonstrated a similar impact with mono-articular exoskeletons affecting force and work contribution about joints with which they do not directly interact [26,41–43]. Our results suggest the decreased knee extensor moment during early leg extension likely reduced acceleration of the thigh segment compared with the control condition, resulting in the thigh gaining less kinetic energy and requiring smaller hip flexor muscle moments to decelerate the thigh at its end range of movement (less negative energy).

Unfortunately, the major limiting factor of this exoskeleton design was that the moment produced was quite small, as the stiffness of the exoskeleton in this study was measured to only be 1–1.3 N/mm, while the Achilles tendon has been previously measured to be 130–200 N/mm [44,45]. As such, mean exoskeleton moment only accounted for 8–11% of mean net plantar flexion moment about the ankle (figures 5 and 6). Furthermore, the exoskeleton moment was relatively constant, as length change was more influenced by knee extension than ankle rotation owing to a longer knee flexor moment arm compared with the ankle moment arm, resulting in minimal recoil during ankle plantar flexion. It should be noted that despite these small forces, there were significant changes in moment produced about the hip and the knee, supporting the idea that bi-articular devices could potentially have a substantial impact on fine-tuning joint mechanics.

Although this initial implementation of the bi-articular exoskeleton produced an unfavourable effect, tuning its design to single out and amplify the brief period of increased biological knee moment may produce a more favourable outcome. Also, a greater stiffness and energy storage capacity might facilitate a greater contribution to net joint moments. The primary factors limiting exoskeleton stiffness in this study were the velcro straps which were at their upper loading limit. Additionally, comfortably attaching exoskeletons to the human body has always been a challenge, as soft tissues must first be loaded before force is applied to the skeleton [46,47]. When our exoskeleton was loaded in the current study, the taught inferior edge of the upper horizontal thigh velcro strap created a high-pressure point that caused discomfort in some participants. Self-adhesive bandages were applied under the exoskeleton to reduce this abrasion, however, thicker compression materials such as foam may provide further improvement. Asbeck *et al*. [24] have designed a textile exoskeleton by anchoring the device to the pelvis, allowing force to be transferred directly to the skeleton with minimal soft tissue loading [24,47], which could be a viable solution. Further tuning could be achieved by employing a stiffer elastic element that was designed to only come under tension later in the movement, when the biological knee moment may be capable of increasing, or alternatively, a nonlinear ‘hardening’ spring might satisfy this purpose. A longer ankle exoskeleton moment arm would also facilitate a greater rate of exoskeleton shortening during plantar flexion, facilitating a larger proportion of energy being delivered to the ankle at the end of push-off. Such a setup would also better match the moment profile of the unassisted ankle and knee joints during jumping to maintain proximal-to-distal joint sequencing. These changes would allow the knee to perform work normally at the start of push-off, then the exoskeleton would resist knee extension when the biological knee extensor moment is able to be increased, after which plantar flexion would be assisted during ankle rotation.

While a previous study observed on average, a 3.1 cm increase in jump height, this was performed with a powered mono-articular ankle device compared with our device which is completely passive. Ben-David *et al*. [1] initially observed a decrease in jump height of roughly 2.2 cm with their passive knee mono-articular exoskeleton, which is comparable with our 1.9 cm decrease in jump height. However, Ben-David *et al*. [1] were subsequently able to increase jump height of their participants through optimization simulations, finding that a much deeper squat depth was required to make use of their exoskeleton, which resulted in an increase of jump height 2.7 cm. Domire & Challis [48] previously theorized that an increase in jump height could be possible by descending to a much deeper squat depth, however, no studies have been able to prove this experimentally in humans. Our current study had a fixed squat depth and thus users were not able to descend to a deeper depth. It was only after the publication by Ben-David *et al*. [1] in 2022 (after our data collection was completed), that it was identified that a deeper squat may be a primary factor in increasing jump height with exoskeletal devices. However, as observed by Ben-David *et al*. without optimization simulation methods to guide our participants towards the alternate optimized jumping technique [1,30], they likely would not have been able to identify if a deeper squat depth could have improved their jump height.

Ben-David *et al*.’s [1] work raises questions over our initial hypothesis that knee extensor moment is limited by joint mechanical constraints during maximal effort tasks, as their study demonstrated that an increase in net knee moment alone was sufficient to increase jump height. However, their device employed a mono-articular spring that substantially assisted knee extension and resisted knee flexion (opposite of this study). Their device actually required positive biological work to be produced during the countermovement, as knee flexor muscles were required in addition to the weight of the participant, to overcome the knee extension moment produced by the exoskeleton. Subsequently, they observed a decrease in the biological knee joint work of 68% and a decreased biological knee extensor moment. The decrease in the biological moment and work may actually reinforce our hypothesis about mechanical constraints limiting knee moments, as the knee in their study likely had a greater capacity to maintain stability of the movement owing to the relatively low submaximal effort required to jump with the device. Based on the results from our study, which indicated that the knee could increase biological moment during mid-push-off, combining of the Ben-David *et al*.’s passive mono-articular knee exoskeleton with an improved version of our passive bi-articular exoskeleton could potentially further increase jump height. A combined device would assist the knee during the start of the push-off (mono-articular knee assistance), then potentially facilitate the knee to increase its work output during the middle of push-off (bi-articular knee resistance), which could then be transferred to the ankle to increase plantar flexion work (bi-articular ankle assistance). However, a greater jump height requires leaving the ground at a higher velocity, which means that joint velocity and muscle shortening velocity will be higher, thus maximal muscle force output will likely be reduced. Future work needs to examine both exoskeletal devices and muscle contraction mechanics, to understand how increasing jump height with a bi-articular exoskeleton will influence the muscles’ ability to produce force.

When developing this exoskeleton, our goal was to design a lightweight device where mass would not be a substantial contributing factor on jump height. However, we do concede that we did not account for the varying individual participant mass’s, while the exoskeleton mass remained relatively constant. Two standard deviations of the average participant mass included in this study gives a range of 53 to 93 kg. Thus, for the lowest mass participants who would have the lightest exoskeleton (0.886 kg), this represents an increase in mass of 1.6%. Alternatively, the highest mass participants who would have the heaviest exoskeleton (1.060 kg), this represents an increase in weight of 1.1%. With only a 0.5% difference in added mass between the lightest and heaviest participants, we believe there would be a negligible effect on their jumping ability across participants. Additionally, the stiffness of the TheraBand tubing was similar across all participants, and thus exoskeleton stiffness was not scaled to the weight of the participant. However, given that stiffness of the exoskeleton was very low (1–1.3 N/mm), compared with the Achilles tendon which has been identified to have a stiffness of 130 N/mm [44,45], we believe any limitation by not scaling stiffness to bodyweight would be negligible.

## 5. Conclusion

This study explored the changes in joint mechanics owing to a bi-articular knee–ankle exoskeleton, which was intended to increase jump height by facilitating greater biological knee extensor moment and net ankle power output. The biological knee joint extension moment had a small increase with the exoskeleton, although only during the mid-late phase of the push-off. However, this did not result in any increase in subsequent ankle joint work output and ultimately resulted in a decrease in jump height. Improvements need to be made to the exoskeleton to optimize how the energy is stored and released, such as introducing a greater slack length, a stiffer elastic element and a longer ankle moment arm. Interestingly, despite the exoskeleton’s low force generation and no direct attachment to the hip, there was a significant increase in work performed about the hip and decrease in work performed about the knee, demonstrating the substantial impact that bi-articular exoskeletons could have on joint mechanics. Bi-articular designs may facilitate devices to be employed with minimal assistance, although this property could also result in a very narrow operating window that may result in a negative impact if the exoskeletal device is not optimized correctly.

## Data Availability

Supplementary material is available online [49]. Individual patient data is included in the supplementary file uploaded with this submission, which includes time-normalized data for key outcome measures, for each participant and condition (Supplementary File 1). Supplementary File 2 includes a video of the exoskeleton being used.
